# Machining Eco-Friendly Jute Fiber-Reinforced Epoxy Composites Using Specially Produced Cryo-Treated and Untreated Cutting Tools

**DOI:** 10.3390/polym16233329

**Published:** 2024-11-27

**Authors:** Mehmet Şükrü Adin, Hamit Adin

**Affiliations:** 1 Besiri OSB Vocational School, Batman University, Batman 72060, Turkey; 2 Faculty of Engineering and Architecture, Batman University, Batman 72100, Turkey

**Keywords:** cryo-treated cutting tool, delamination, eco-friendly composite, machining optimization, surface roughness, statistical analysis

## Abstract

In recent years, consumers have become increasingly interested in natural, biodegradable and eco-friendly composites. Eco-friendly composites manufactured using natural reinforcing filling materials stand out with properties such as cost effectiveness and easy accessibility. For these reasons, in this research, a composite workpiece was specially manufactured using eco-friendly jute fibers. Two cost-effective cutting tools were specially produced to ensure high-quality machining of this composite workpiece. One of these specially manufactured cutting tools was subjected to DC&T (deep cryogenic treatment and tempering) processes to improve its performance. At the end of the research, when the lowest and highest Fd (delamination factor) values obtained with DC&T-T1 and T1 cutting tools were compared, it was observed that 5.49% and 6.23% better results were obtained with the DC&T-T1 cutting tool, respectively. From the analysis of the S/N (signal-to-noise) ratios obtained using Fd values, it was found that the most appropriate machining parameters for the composite workpiece used in this investigation were the DC&T-T1 cutting tool, a 2000 rev/min spindle speed and a 100 mm/min feed rate. Through ANOVAs (analyses of variance), it was discovered that the most significant parameter having an impact on the Fd values was the spindle speed, with a rate of 53.01%. Considering the lowest and highest Ra (average surface roughness) values obtained using DC&T-T1 and T1 cutting tools, it was seen that 19.42% and 16.91% better results were obtained using the DC&T-T1 cutting tool, respectively. In the S/N ratio analysis results obtained using Ra values, it was revealed that the most appropriate machining parameters for the composite workpiece used in this investigation were the DC&T-T1 cutting tool, a 2000 rev/min spindle speed and a 100 mm/min feed rate. In the ANOVAs, it was revealed that the most significant parameter having an effect on the Ra values was the feed rate at 37.86%.

## 1. Introduction

In today’s contemporary manufacturing industries, synthetic fiber-reinforced composites are commonly employed due to numerous superiorities such as their light weight, high strength and corrosion resistance [1,2]. The most prominent of these synthetic fiber-reinforced composites, which are employed in many different fields from aviation to the automobile industry, are GFR (glass fiber-reinforced) and CFR (carbon fiber-reinforced) composites [3,4]. However, since CFR and GFR composites are not natural, they can cause significant environmental problems after use. For this reason, in recent years, consumers have become increasingly interested in natural, biodegradable and eco-friendly composites [3,5]. Eco-friendly composites manufactured using natural reinforcing filling materials stand out with properties such as cost effectiveness and easy accessibility [3]. In manufacturing industries, some of the most well-known natural reinforcing fillers as alternatives to synthetic fibers are jute, flax, hemp, kenaf, bamboo and pineapple. Jute fibers, which are employed extensively in various industrial areas (for example, packaging, carpet base fabric and wall coverings), have relatively recently begun to be used as natural reinforcing filling material in the manufacture of composite materials [1,3]. Jute fibers materials, whose mechanical properties vary depending on the production methods, are quite strong but have low elongation and elasticity values [6].

Composites produced for different manufacturing industries are generally manufactured with dimensions very close to their final use. However, depending on where these materials are used, machining operations like drilling, trimming or milling are required to combine them with materials of the same type or those with different properties. Among these operations that require high-quality machining, drilling operations stand out proportionally as the most significant [7,8]. Since the quality of the holes drilled in composite materials directly affects the functions performed by fasteners, such as the rivets and bolts used for joining, the lack of integrity of the created structure can result in catastrophic failure [7]. Accordingly, intensive research is necessary to carry out high-quality hole-drilling operations in new and different composite materials. The drilling process with cutting tools is varying and complex, as it is affected by many different parameters, such as cutting tool geometry and type. Therefore, the high-quality machining of materials with different properties depends on the suitability of these parameters for the cutting tool [9]. Additionally, it is among the priorities of the manufacturing industries that the cutting tools preferred for hole-drilling operations are easily obtainable and cost effective, just like the workpiece. Due to these demands, HSS (High-Speed Steel) tools are preferred at high rates globally (40% of all cutting tools) [10,11]. However, when compared to expensive new-generation cutting tools such as ceramic and carbide types, it has been understood that HSS cutting tools need to be developed with regard to features such as hardness, wear resistance and heat resistance [12,13]. Previous studies have shown that these properties of HSS cutting tools can be improved [14]. All metal materials have different properties depending on their different chemical composition and microstructure. Therefore, when it is desired to improve the properties of metals, it is necessary to intervene either in their microstructure or in their chemical composition [15]. Since intervention in chemical composition is a difficult process, intervention in microstructures is preferred. One of the most commonly used methods for intervention in microstructures is conventional heat treatment [12]. However, after conventional heat treatment is applied to cutting tools, undesirable soft phases (austenite) remain in the microstructure. These undesirable soft phases, called retained austenite, negatively affect a cutting tool’s life [15]. In order to solve this problem, recently, cryogenic treatment (sub-zero process) has been applied to cutting tools. With cryogenic treatment, which causes a change in the cutting tool’s microstructure, the soft retained austenite phase transforms into a harder martensitic phase (also with a decrease in the volume of carbides in the microstructure and a more homogeneous distribution of them), resulting in significant increases in the performance of the cutting tool [16]. Cryogenic treatments applied to cutting tools are generally carried out in two different ways, expressed as deep cryogenic treatment (below −125 °C) and shallow cryogenic treatment (between −50 °C and −100 °C), depending on the applied sub-zero temperature values [17]. The main reason for applying these two different treatments is that the weight percentage of retained austenite in the microstructure decreases at different rates [18]. As a matter of fact, when the retained austenite rates in the microstructures of tools exposed to deep cryogenic treatment, shallow cryogenic treatment and conventional heat treatment are examined, it is observed from the literature that they are 5.1–2.7%, 6.5–5.1% and 6.5%, respectively [18,19,20]. By decreasing or even eliminating the proportion of retained austenite in their microstructures, significant increases occur in the toughness, hardness and wear resistance of the tools. Moreover, cryogenic treatment is reported to better the electrical and thermal conductivities of cutting tools [21]. Consequently, all these desired, favorable improvements positively affect a cutting tool’s performance [15,22].

In the literature review regarding the machining of composites, it was seen that the majority of studies were related to synthetic fiber-reinforced composites. However, recently, there has been great tendency toward eco-friendly composites, since synthetic fiber-reinforced composites are not natural and cause significant environmental problems. The aim of this study is to produce an eco-friendly fiber-reinforced composite and to determine the most optimum cutting tool for high-quality machining of this composite workpiece. In this context, in this research, the composite workpiece was specially manufactured using eco-friendly jute fibers. Additionally, it has been observed that the studies in the literature are generally carried out using cutting tools having standard geometric dimensions (clearance, helix and point angles), and therefore, composite workpieces cannot be machined with high quality. For all these reasons, within the scope of this research, two cutting tools were specially manufactured according to the geometric dimensions determined in light of previous studies. Moreover, one of these specially manufactured cutting tools was subjected to deep cryogenic treatment to improve its performance. Finally, in order to make the research cost effective and evaluate the results obtained with high accuracy, the Taguchi method was employed; additionally, regression and ANOVA analyses were also performed.

## 2. Materials and Methods

### 2.1. Manufacturing of the Jute Fiber-Reinforced Composite Material

**Figure 1:**
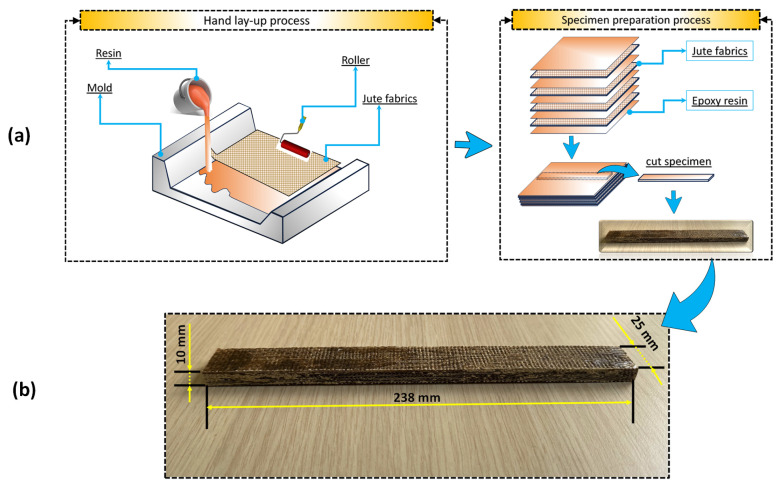
(**a**) Hand lay-up and specimen preparation processes; (**b**) dimensions of the composite workpiece.

In this research, a specially manufactured jute fiber-reinforced epoxy composite plate was used as the workpiece. ARC-152 brand epoxy resin (product of the ARC Marine company, Singapore) and jute fibers (woven fabrics) were used for the production of the composite workpiece. ARC-152 brand epoxy resin (also curing agents), which is highly compatible with different fiber types (specified in the catalogue), was chosen. In line with the recommendation in its catalogue, the epoxy/accelerator ratio was applied as 4/1 by volume in order to provide the highest strength. Before starting the composite production, jute fibers (product of Bonanza Jute Composite & Diverse Factory, Dhaka, Bangladesh) were dried in an oven at 55 °C (approximately 24 h) to ensure that they were moisture-free. Then, jute fibers were cut into dimensions of 295 mm × 295 mm in the size of the mold. Additionally, polyvinyl alcohol (PVA) was applied to the floor to prevent adhesion, and after the PVA was completely dry, the production process was started using the hand-laying method. Figure 1a shows the hand lay-up process and the materials used.

As depicted in Figure 1a, where the hand lay-up and specimen preparation processes are given, one layer of epoxy resin and one layer of jute fiber (dust cleaned) were laid on top of each other and applied thoroughly with the help of rollers. Following the processes, a composite plate consisting of 12 layers was produced. In order for the adhesive in the produced composite workpiece to cure, it was kept at room temperature for approximately 25 h, in line with the recommendations in the catalogue. After the curing process of the composite workpiece was completed, it was precisely cut with a water jet. The main reason for cutting using a water jet is to prevent any delamination that may occur in the composite workpiece. Figure 1b shows the dimensions of the composite workpiece (238 mm × 25 mm × 10 mm) that was cut with a water jet and made ready for machining.

### 2.2. Manufacturing of Cutting Tools

**Figure 2:**
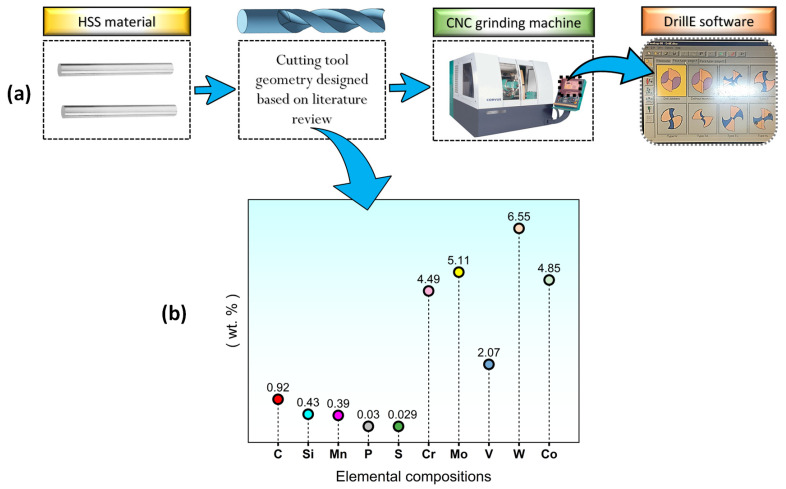
(**a**) Flow chart of cutting tool manufacturing; (**b**) elemental composition of HSS material.

In this research, instead of cutting tools with standard dimensions, special cutting tools (2 drill bits) were manufactured according to the dimensions recommended in the literature. Moreover, in order to make the research more cost-effective, High-Speed Steel (HSS), which is not very difficult to procure, was used in the manufacturing of the cutting tools [23,24]. In Figure 2, the elemental composition of the HSS material used in the manufacturing of cutting tools is depicted.

As is known, in the aerospace industry, where high-quality machining is carried out, drills with diameters between 5 and 10 mm are generally used in the machining of composite materials [25,26,27]. That is why, within the scope of this research, the diameters of the specially manufactured cutting tools were determined as 8 mm. Likewise, in light of previous research studies (related to composite materials), the clearance angle, helix angle and point angle of the cutting tools were selected as 10°, 30° and 135°, respectively [26,27]. For the purpose of manufacturing cutting tools from rod-shaped HSS materials (with an 8 mm diameter and 63 mm length), a CNC Schneeberger Corvus GDS brand grinding machine and DrillE software that was included with this machine were used. In Figure 2a, the flow chart of the manufacturing of cutting tools is depicted.

### 2.3. Deep Cryogenic and Tempering Treatments

Within the scope of this research, cryogenic treatment (CT) was also applied to one of the specially manufactured cutting tools. With the CT process, which causes a change in the microstructure of cutting tools, the soft retained austenite phase transforms into a harder martensitic phase (also with the decrease in the volume of carbides in the microstructure and more homogeneous distribution of them), resulting in significant increases in the performance of the cutting tool [16,21,24]. As mentioned above, CT processes have significant effects on materials and should be carefully determined according to the materials used in the manufacturing of cutting tools [16,24]. In this context, in light of previous studies, the CT process parameters, such as the cooling/heating rate, soaking temperature and soaking time, applied to the HSS cutting tool were carefully determined. The values of these carefully determined parameters are 0.5 °C/min (cooling/heating rate) [28], −196 °C (soaking temperature for deep cryogenic treatment (DC)) [22] and 24 hours (soaking time) [29], respectively. Following the DC process, the cutting tool was also tempered (for 2 h at 200 °C [30]), as recommended in the literature.

**Figure 3:**
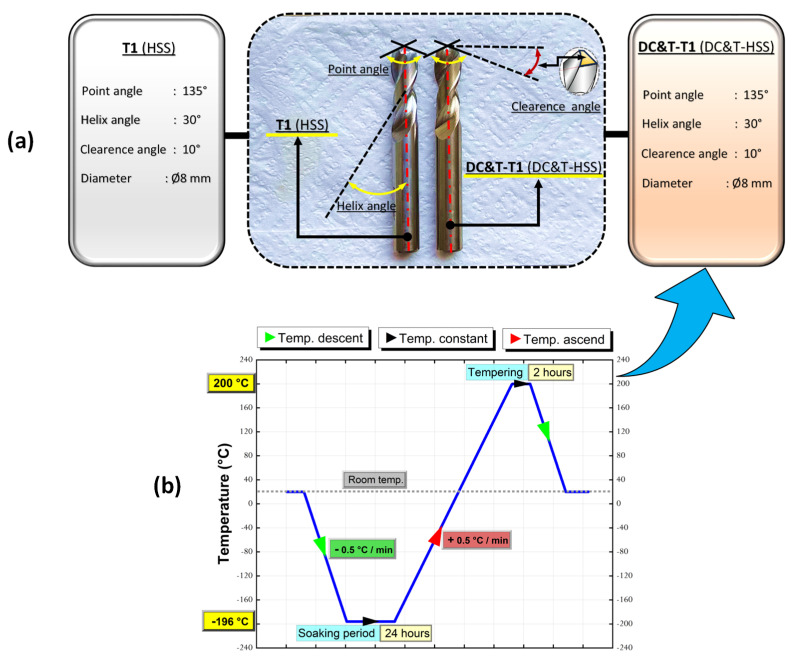
(**a**) Geometric dimensions of the DC&T-T1 and T1 cutting tools; (**b**) stages of the DC and T processes applied to the cutting tool.

In this current research, the cutting tool without treatment and the cutting tool with DC and tempering (T) processes are expressed as T1 and DC&T-T1, respectively. The stages of DC (using liquid nitrogen) and T processes applied to the cutting tool are depicted in Figure 3.

In Figure 3a, the geometric dimensions of the DC&T-T1 and T1 cutting tools are depicted. As shown in Figure 3a, after the DC&T processes, the color of the DC&T-T1 tool turned into a color close to orange.

### 2.4. Using the Taguchi Method for Machining

Nowadays, most contemporary manufacturing industries utilize many optimization methods. Of these very different industries, the Taguchi optimization method is one of the most popular optimization methods. Among the main reasons for the popularity of this method, it can be said that this method has advantages such as being easily applicable, systematic and efficient. Using this optimization method, the number of experiments can be minimized, and the most suitable processing parameters can be found, and thus, cost-effective solutions can be offered [31,32]. For these positive reasons, the Taguchi optimization method was used in this research.

In this present research, the “smaller is better” approach was adopted, since the lowest delamination factor value and surface roughness value were desired. In accordance with this approach, Equation (1), presented below, was utilized to calculate S/N (signal-to-noise) ratios. The “*y_i_*” and “*n*” parameters given in Equation (1) are expressed as the “*i*-th number of observations” and the “number of observations” [31,32].

(1) $$\mathrm{Smaller}\mathrm{is}\mathrm{better}:\;\;\;\;S/N=-10\;log(\frac{1}{n}\sum_{i=1}^{n}y_{i}^{2})$$

Within the scope of this investigation, the Minitab statistical program was employed for high-accuracy analysis of the experimental results obtained. The cutting parameters applied in this research were determined as a consequence of intensive literature searches, and different values were selected by taking into account the lower and upper values in order to make the research unique. In Table 1, the machining parameters determined for this study are given.

In this investigation, the L18 (2^1^ × 3^2^) mixed-level design (based on machining parameters and levels) was selected to obtain the optimum experimental results. Additionally, in this current research, analysis of variance, known as ANOVA, was carried out at 95% confidence and 5% significance levels to find the percentage contribution values of the machining parameters [31,32]. Furthermore, regression equations were obtained as a consequence of regression analyses and then compared with the experimental results.

### 2.5. Machining Experiments

**Figure 4:**
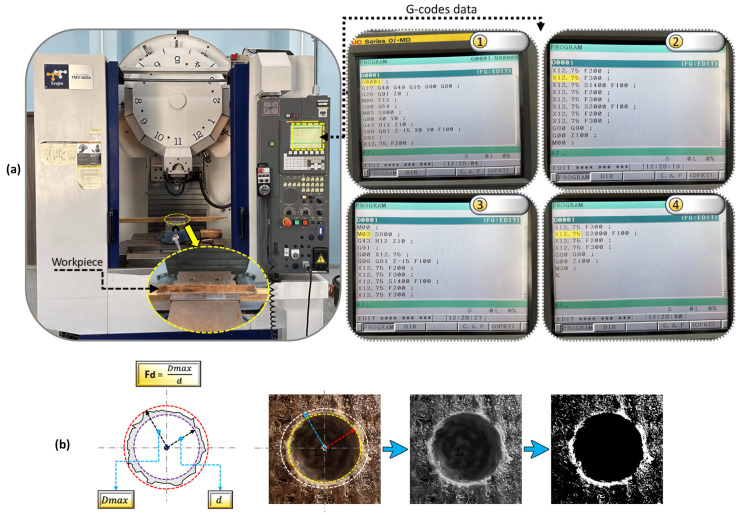
(**a**) G-code screenshots and CNC vertical machining center; (**b**) Fd measurement.

In this experimental research, a CNC Topper Tongtai TMV-610A brand vertical machining center was employed for the machining experiments (hole drilling) conducted under dry conditions. In the machining experiments, holes were drilled into the composite workpiece based on the Taguchi (L18) experimental design, using specially manufactured 8 mm diameter DC&T-T1 and T1 cutting tools. In Figure 4, the CNC vertical machining center and G-codes used in this research are shown.

In Table 2, the machining parameters and levels determined based on Taguchi L18 are given. As seen in Table 1 and Table 2, the specially manufactured composite workpiece was machined at different spindle speeds (800, 1400 and 2000 rev/min) and feed rates (100, 200 and 300 mm/min) using two specially manufactured cutting tools (DC&T-T1 and T1).

In this research, measurements were made using a Hubstein HTC-1 brand device, since the sensitivity (i.e., negative effects on the experimental results) of composite materials to ambient temperature and relative humidity was known from previous studies [6]. As a consequence of the measurements, the average values of relative humidity and ambient temperature were found to be 55% and 24 °C, respectively.

### 2.6. Ra and Fd Measurements

In contemporary manufacturing industries, it is desired that the hole surfaces of drilled samples be of high quality. In line with this aim, specimens with the lowest average surface roughness (Ra) value are accepted as priority. The reasons why the lowest Ra values are preferred include the much higher lifetime and performance of the specimens [9,33]. Due to the importance of Ra values of hole surfaces, in this research, measurements were made five times from different locations per hole using a Hommel Tester T500 brand device, and their averages were calculated. Because of the delamination that frequently occur during the drilling of composite workpieces, almost 60 percent of the specimens produced in manufacturing industries fail to receive acceptance (in other words, they are rejected) [34]. For this reason, in the present research, any delamination occurring in the composite workpiece was specifically investigated. In this context, images of all holes were first taken using a Leica DM 750P branded optical microscope, and then the delamination factor values were calculated precisely with the help of ImageJ software. As seen in Figure 4b, the formula used to calculate the Fd value is shown on both a technical drawing and a real image taken. As depicted in Figure 4b, “Fd”, “Dmax” and “d” in the formula (Fd = Dmax × d^−1^) represent the “delamination factor”, “the maximum diameter of the delamination area” and “the diameter of the cutting tool (drill)”, respectively [7].

## 3. Results and Discussion

### 3.1. Delamination Factor (Fd)

**Figure 5:**
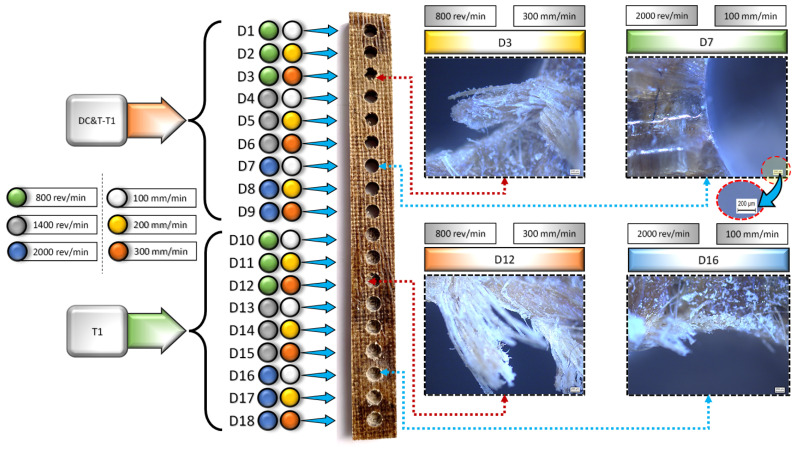
Machined composite workpiece and its images (magnification: 200 µm) taken with a microscope.

Nowadays, although the majority of composite materials are manufactured in the geometric structure closest to their final use shape, machining is required to mechanically combine them with other materials in the places of use. In order to meet this need (for the use of fasteners such as rivets and bolts), one of the most used methods (with a rate as high as 40%) in the machining of composite materials is the hole-drilling method [34,35,36]. However, due to the anisotropic and heterogeneous nature of composite materials, many types of damage (such as fiber pullout, delamination and hole shrinkage) occur during machining operations. Among these damage types, delamination damage (also known as layer separation or fiber breakage) is the most undesirable type of damage, as it influences the strength and service life of the composite material most negatively [34,35,37]. As a matter of fact, approximately 60% of the composite parts machined in the manufacturing industry fail to pass sample acceptance tests due to such damages [35,38]. Due to such negative consequences of delamination damages occurring during hole-drilling operations, within the scope of this experimental research, delamination damages occurring in the specially produced composite workpiece were specifically investigated. In this context, images of all holes were first taken using a Leica DM 750P branded optical microscope, and then the delamination factor (Fd) values were calculated precisely with the help of ImageJ software. In modern manufacturing industry, it is desired that the Fd values resulting from the drilling of composite materials should be 1 (i.e., without delamination) or as close to 1 as possible [39]. As is known, Fd values are influenced by numerous factors, for instance, the geometry of the cutting tool, properties of the cutting tool, machining parameters and properties of the composite workpiece [9]. That is why each of these factors needs to be examined separately. As displayed in Figure 5, the image of the composite workpiece machined at different parameters and levels based on Taguchi DOE with DC&T-T1 and T1 cutting tools as well as the images taken with a microscope are given.

When the images presented in Figure 5 are controlled, it is observed that the delamination occurring at the edges of the hole entrance changes with the influence of different cutting tools (DC&T-T1 and T1) and machining parameters. For this reason, within the scope of this research, each parameter affecting Fd values was meticulously investigated.

**Figure 6:**
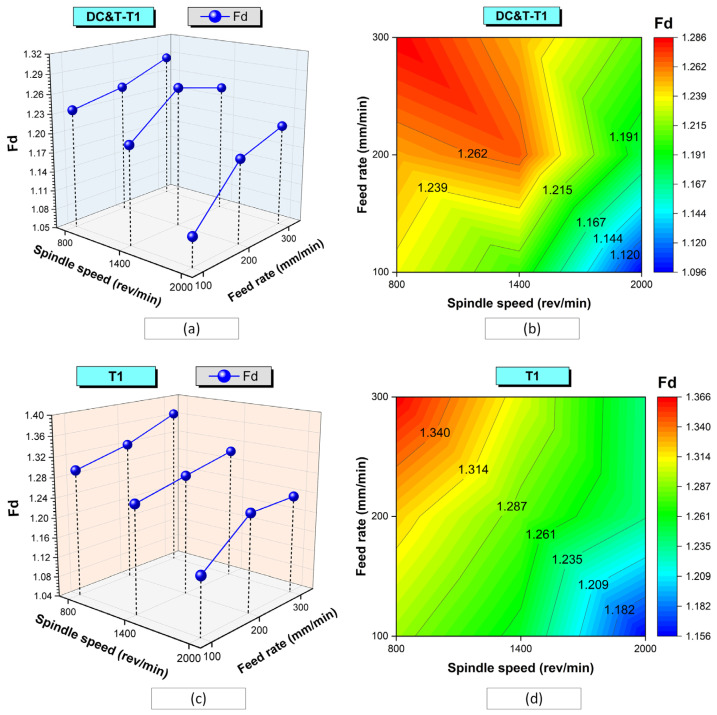
Effects of cutting tools (DC&T-T1 and T1) and machining parameters on Fd values: (**a**,**b**) for DC&T-T1; (**c**,**d**) for T1.

In Figure 6, the impacts of cutting tools (DC&T-T1 and T1) and machining parameters on Fd values are shown comparatively. As displayed in Figure 6, due to the Taguchi DOE applied within the scope of the study, the influences of machining parameters on Fd values are shown separately according to DC&T-T1 and T1 tools.

As depicted in Figure 6a, the highest Fd value occurring at the hole edges of the composite workpiece machined with the DC&T-T1 cutting tool turned out to be 1.286 (see Figure 5, D3). This highest Fd value was obtained using the DC&T-T1 cutting tool at the applied machining parameters of an 800 rev/min spindle speed and 300 mm/min feed rate. On the other hand, as exhibited in Figure 6a, the lowest Fd value occurring at the hole edges of the composite workpiece machined with the DC&T-T1 cutting tool turned out to be 1.096 (see Figure 5, D7). This lowest Fd value was obtained using the DC&T-T1 cutting tool at the applied machining parameters of a 2000 rev/min spindle speed and 100 mm/min feed rate. When the highest Fd value (1.286) and the lowest Fd value (1.096) were compared, it was found that the highest Fd value was 17.34% higher than the lowest Fd value. As a matter of fact, when the graph (counter-plot) given in Figure 6b is checked, it is noticed that the highest Fd values are concentrated (red-colored area) around the machining parameters of an 800 rev/min spindle speed and 300 mm/min feed rate. Additionally, when the graph (counter-plot) given in Figure 6b is examined, it is noticed that the lowest Fd values are concentrated (blue-colored area) around the machining parameters of a 2000 rev/min spindle speed and 100 mm/min feed rate.

Moreover, as depicted in Figure 6c, the highest Fd value occurring at the hole edges of the composite workpiece machined with the T1 cutting tool was found to be 1.366 (see Figure 5, D12). This highest Fd value was obtained using the T1 cutting tool at the applied machining parameters of an 800 rev/min spindle speed and 300 mm/min feed rate. On the other hand, as seen in Figure 6c, the lowest Fd value occurring at the hole edges of the composite workpiece machined with the T1 cutting tool was found to be 1.156 (see Figure 6, D16). This lowest Fd value was obtained using the T1 cutting tool at the applied machining parameters of a 2000 rev/min spindle speed and 100 mm/min feed rate. When the highest Fd value (1.366) and the lowest Fd value (1.156) were compared, it was found that the highest Fd value was 18.17% higher than the lowest Fd value. As a matter of fact, when the graph (counter-plot) given in Figure 6d is checked, it is noticed that the highest Fd values are concentrated (red-colored area) around the machining parameters of an 800 rev/min spindle speed and 300 mm/min feed rate. Additionally, when the graph (counter-plot) given in Figure 6d is checked, it is noticed that the lowest Fd values are concentrated (blue-colored area) around the machining parameters of a 2000 rev/min spindle speed and 100 mm/min feed rate.

**Figure 7:**
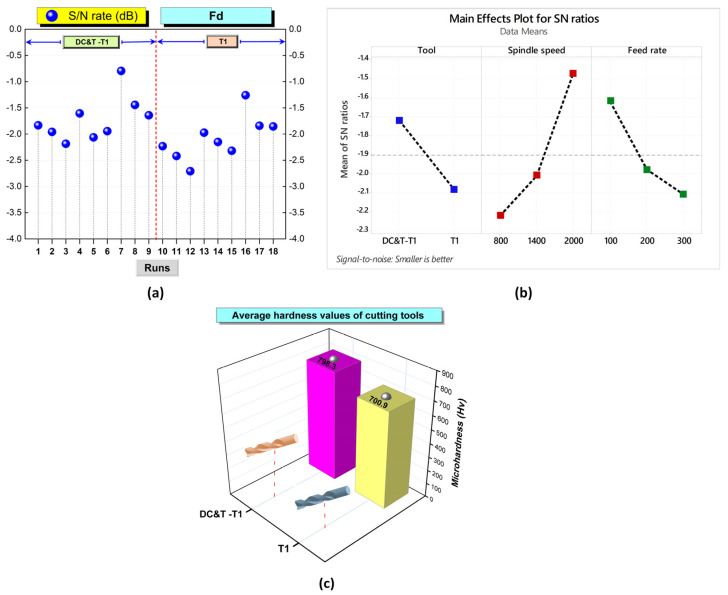
(**a**) Based on Fd values, S/N ratios for each hole. (**b**) Basic effects plot for Fd values. (**c**) Average hardness values of cutting tools.

As a consequence, as seen in Figure 5 and Figure 6, when the Fd values at the edges of the holes in the composite workpiece machined (with the same parameters and levels) using DC&T-T1 and T1 cutting tools were compared, it was observed that better results (i.e., lower Fd values) were obtained with the DC&T-T1 cutting tool. Moreover, when the lowest and highest Fd values obtained with the DC&T-T1 and T1 cutting tools were compared, it was seen that 5.49% and 6.23% better results (i.e., lower Fd values) were obtained with the DC&T-T1 cutting tool, respectively. Furthermore, in this current research, detailed analyses were carried out using the Minitab program in order to better evaluate the Fd values of the holes in the composite workpiece machined using DC&T-T1 and T1 tools. With the help of the Minitab program, S/N ratios were calculated based on the Fd values obtained from the holes and are shown in Figure 7a.

**Figure 8:**
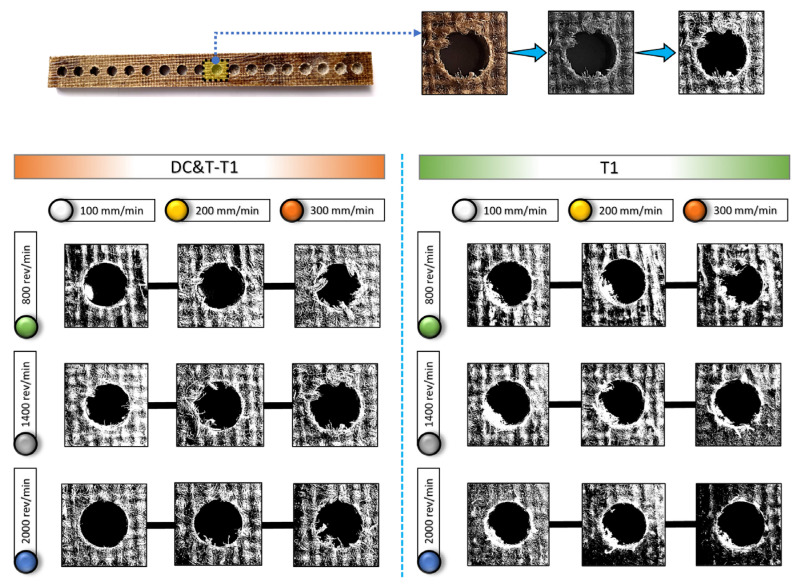
Delamination at different machining parameters according to the cutting tools used in the study.

As seen in Figure 6 and Figure 7, the highest S/N ratio (using the Fd value obtained from the hole drilled with the DC&T-T1 cutting tool) was calculated as −0.79621 dB (for an Fd value of 1.096). Additionally, the lowest S/N ratio (using the Fd value obtained from the hole drilled with the T1 cutting tool) was calculated as −2.70901 dB (for an Fd value of 1.366). Moreover, as illustrated in Figure 7, although both cutting tools show similar trends depending on the changes in parameters and levels, the DC&T-T1 cutting tool appears to have clear superiority (since a high S/N ratio is desired [31,32]). In this research, in order to better understand the influences of the machining parameters (cutting tool, spindle speed and feed rate), the main effects graph was obtained using S/N ratios and is illustrated in Figure 7b. Once the main impacts on the plot presented in Figure 7b are checked, it is clearly observed that the better results (i.e., lower Fd values) are obtained with the DC&T-T1 cutting tool. Additionally, as illustrated in the basic effects plot (Figure 7b), Fd values are positively affected (i.e., lower Fd values) as the spindle speed increases, while Fd values are negatively affected (i.e., higher Fd values) as the feed rate increases. Furthermore, when the analysis of the S/N ratios (Figure 7b) obtained using Fd values was examined, it was revealed that the most appropriate machining parameters (in order to obtain the lowest Fd values) for the composite workpiece employed in this investigation were that of the DC&T-T1 cutting tool, a 2000 rev/min spindle speed and 100 mm/min feed rate, respectively. Additionally, within the scope of this current research, separate images of each hole were taken to better understand the effects of the parameters and levels applied for the machining of the specially manufactured composite workpiece. Figure 8 displays the delamination (for each hole) occurring at different machining parameters according to the cutting tools used in the study. Delamination at the edges of the holes machined with the DC&T-T1 cutting tool is found to be less than that at the edges of the holes machined with the T1 cutting tool, as shown in Figure 8. However, when the holes drilled with both cutting tools were examined, it was observed that similar drilling-induced damages occurred. Additionally, it was determined that delamination increased with an increasing feed rate at a constant spindle speed in machining operations performed with both cutting tools. On the other hand, it was found that delamination reduced when the spindle speed was increased at a constant feed rate in machining operations performed with both cutting tools. Moreover, when the holes machined with both cutting tools (DC&T-T1 and T1) were inspected, it was seen that delamination and drilling-induced damages started to decrease significantly as the optimal cutting parameters were approached. As a matter of fact, this situation is better seen in the cutting parameters where a spindle speed of 2000 rev/min and a feed rate of 100 mm/min are applied.

As is known, by reducing or completely eliminating the remaining austenite ratio in the microstructure of cutting tools (HSS tools), significant increases (i.e., betterments) in the toughness, hardness and wear resistance of the cutting tools occur. Moreover, cryogenic treatment is reported to better the electrical and thermal conductivities of cutting tools [21,23]. Consequently, all these desired, favorable improvements positively affect the performance of cutting tools [15,22]. In this current research, microhardness measurements were conducted with the help of a Matsuzawa brand device in order to understand the effects of the DC&T processes applied to the specially manufactured T1 tool. In Figure 7c, the average hardness values (arithmetic average of measurements taken from five different points) obtained as a result of separate measurements of the DC&T-T1 and T1 cutting tools are illustrated. As illustrated in Figure 7c, the average hardness value (798.3 HV) of the cutting tool (DC&T-T1) subjected to DC&T processes was found to be 13.89% higher than the average hardness value (700.9 HV) of the untreated T1 cutting tool. It was observed that these results were in harmony with the results (betterment in properties such as wear resistance, hardness, toughness and thermal conductivity of cutting tools) of previous studies [14,21,40]. Moreover, with the effect of these positive improvements in the cutting tool, the Fd values are positively affected due to less cutting force (i.e., since it cuts the composite workpiece more easily) being required [41,42]. Consequently, with the positive effect of DC&T processes applied to the cutting tool (DC&T-T1), the performance of the cutting tool was also positively affected.

As seen in Figure 5 and Figure 8, it is observed that delamination increases as the feed rates increase (from 100 mm/min to 300 mm/min) in the holes drilled with both cutting tools (DC&T-T1 and T1). However, it was revealed that delamination was lower (as desired) in the drilling operations performed with low feed rates. This can be explained by the fact that less damage occurs on the hole edges due to the better active cutting process (during the interaction between the cutting tool and the composite workpiece) in drilling operations performed at low feed rates. Moreover, since the thermal conductivity of the composite workpiece employed in this research is quite low [6,41], the temperature generated during the interaction between the composite workpiece and the cutting tool cannot move away from the area where the cutting process takes place; thus, the composite workpiece softens and positively affects the cutting process [41,43]. Furthermore, since the interaction time between the composite workpiece and cutting tool rises due to the low feed rate, it becomes possible to eliminate deficiencies in areas where the cutting process cannot be fully performed. As a matter of fact, when similar studies (studies carried out on composite workpieces) in the literature were inspected, it was revealed that the Fd values (i.e., less delamination) obtained in drilling operations performed with low feed rates were parallel to the Fd values obtained within the scope of this research [44,45]. On the other hand, as the feed rate increases, the cutting process cannot be fully performed owing to the rise in the volume of the chip that the cutting tool tries to remove from the composite material, which has a negative effect on Fd values. Here, the cutting tool dives into the composite material faster owing to the rise in the feed rate, but since the cutting process cannot be performed fully, it starts to pull and tear the composite material instead of cutting it. Moreover, as displayed in Figure 5 and Figure 8, due to the rapid dive of the cutting tool into the composite material, the width of the uncut fibers and delamination at the hole entrances increase, and the integrity of the composite material starts to deteriorate [41,43,44]. On the other hand, as displayed in Figure 5 and Figure 8, it is observed that delamination decreases as the spindle speed increases (from 800 rev/min to 2000 rev/min) at a constant feed rate in the holes drilled with both cutting tools (DC&T-T1 and T1). This can be explained by the fact that less damage occurs on the hole edges due to the better active cutting process (during the interaction between the composite workpiece and the cutting tool) in drilling operations performed at high spindle speeds. Furthermore, due to the positive effect of the high spindle speed, high temperatures occur on the upper surface of the composite material; consequently, the cutting tool performs the active cutting process more easily, and thus, the damages caused by the drilling process begins to occur at minimum values. As a matter of fact, when similar studies (studies carried out on composite workpieces) in the literature were inspected, it was noticed that the Fd values (i.e., less delamination) obtained in drilling operations performed with high spindle speeds were parallel to the Fd values obtained within the scope of this research [2,41,43,46,47].

#### 3.1.1. ANOVA (Analysis of the Variance) and Regression Analyses Based on Fd Values

**Figure 9:**
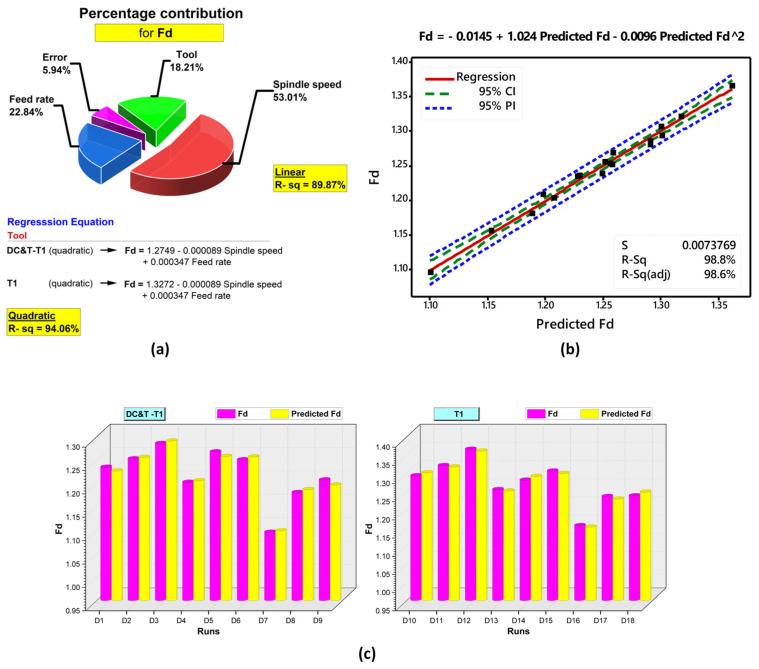
(**a**) ANOVA results and regression equations for Fd. (**b**) Comparison of experimental Fd values and predicted Fd values. (**c**) Fd results vs. predicted Fd values.

In this current research, analysis of variance, known as ANOVA, was carried out at 95% confidence and 5% significance levels to find the percentage contribution values (in order to better understand the impact of each factor on the Fd values) of the cutting tools and cutting parameters [31,32]. In Figure 9a, the results of the ANOVA conducted according to the Fd values are visualized. As displayed in Figure 9a, when the ANOVA results acquired according to the Fd values obtained for each hole within the scope of this experimental study were examined, it was discovered that the most significant parameter having an impact on the Fd values was the spindle speed, with a rate of 53.01%. Additionally, the percentage contribution rates of other parameters affecting the Fd values turned out to be 22.84% (feed rate) and 18.21% (tool), respectively.

As a matter of fact, when the pictures of the hole edges given in Figure 5 and Figure 8 are inspected, it is observed that they confirm the ANOVA results. Additionally, as illustrated in Figure 9a, regression analyses were carried out based on the Fd values obtained within the scope of this research. With regression analysis, the correlation between process parameters and the responses of any machining process is analyzed in detail, and high-accuracy regression equations are developed [31,48]. As depicted in Figure 9a, as a consequence of the analyses performed according to the Fd values, equations independent of each other for DC&T-T1 and T1 cutting tools were formulated as quadratic and linear models. When the regression coefficients (R-sq, that is, R2) indicating the effectiveness of the developed models [32] were examined (Figure 9a), it was seen that the R2 value (94.06%) of the quadratic model was considerably higher than the R2 value (89.87%) of the linear model. The high regression coefficients (approximately 90% and above) obtained show that the equations developed for the DC&T-T1 and T1 cutting tools are highly successful in predicting Fd values [31,32]. In Figure 9b, the comparison of the Fd values estimated using the highly successful quadratic regression model with the experimentally obtained Fd results is depicted. When the graphic (Figure 9b) comparing the experimental results with the predicted values is visually examined, it is discovered that the outcomes (black squares) are gathered quite close to the red line (regression line). Considering these achieved results, it is found that the developed model is highly successful and reliable in predicting Fd values [32,49]. Furthermore, each experimental result and the predicted value are compared separately (with the aim to demonstrate the high success of the developed model) and displayed in Figure 9c. When the experimental Fd results and predicted Fd values given in Figure 9c are visually controlled, it is seen that the experimental Fd results and predicted Fd values are equal or quite close to each other. In light of these results, it is clearly seen that the developed model (for the purpose of predicting Fd values) is highly reliable and successful.

### 3.2. Surface Roughness

**Figure 10:**
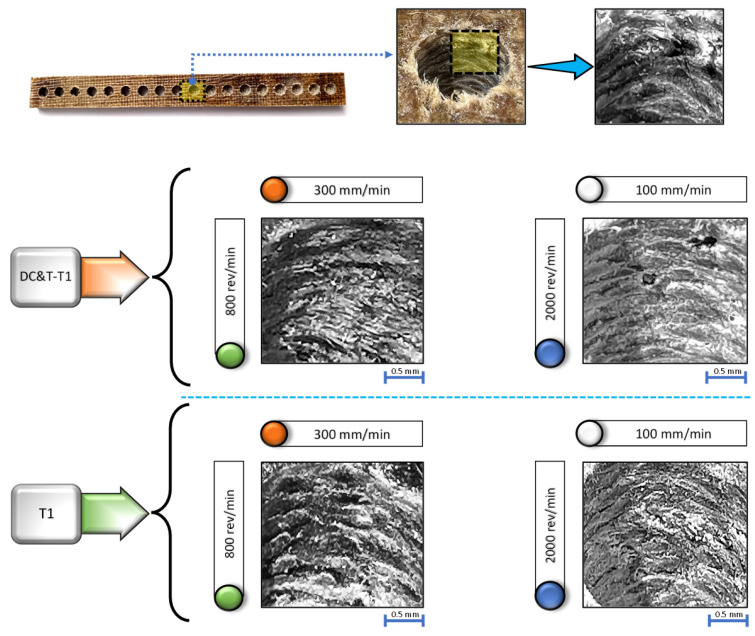
Surface roughness at different machining parameters.

As is known, the surface roughness of a machined workpiece is considered as one of the most important favorable conditions (for the place to be used as a component) by manufacturing industries, as it directly affects its performance [33,36]. Therefore, as in all contemporary manufacturing industries, the average surface roughness (Ra) values of the drilled holes were also precisely measured in this current research [33,37]. In contemporary manufacturing industries, it is desired that the hole surfaces of drilled samples be of high quality. In line with this aim, specimens with the lowest Ra value are accepted as priority. The reasons why the lowest Ra values are preferred include the much higher lifetime and performance of the specimens [9,33]. Due to the importance of the Ra values of the hole surfaces, in this research, measurements were taken five times from different locations per hole, and the averages of all the results obtained were computed. As is known, Ra values are influenced by a number of factors, such as the geometry of the cutting tool, properties of the cutting tool, machining parameters and composite workpiece properties [9]. That is why each of these factors needs to be examined separately. As displayed in Figure 10, the image of the composite workpiece machined at different parameters and levels based on the Taguchi DOE with DC&T-T1 and T1 cutting tools as well as the images taken from holes are given.

When the images given in Figure 10 are examined, it is seen that the surface roughness occurring at the holes change with the influence of different cutting tools (DC&T-T1 and T1) and machining parameters. Accordingly, within the scope of this research, each parameter affecting Ra values was meticulously investigated.

**Figure 11:**
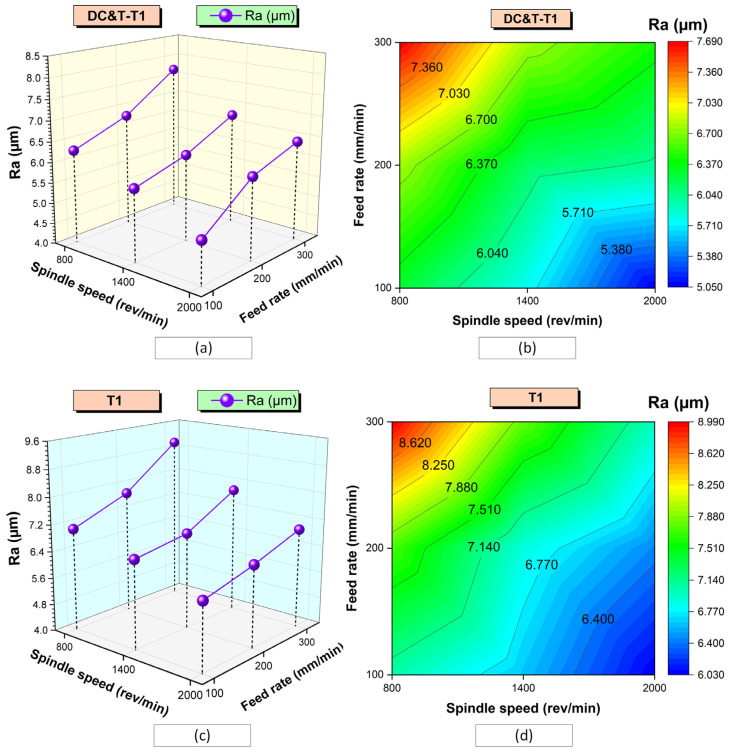
Effects of cutting tools (DC&T-T1 and T1) and machining parameters on Ra values; (**a**,**b**) DC&T-T1 tool, (**c**,**d**) T1 tool.

In Figure 11, the impacts of cutting tools (DC&T-T1 and T1) and machining parameters on Ra values are shown comparatively. As displayed in Figure 11, due to the Taguchi DOE applied within the scope of the study, the impacts of machining parameters on the Ra values are shown separately according to the DC&T-T1 and T1 tools.

As illustrated in Figure 11a, the highest Ra value occurring in the holes drilled in the composite workpiece machined with the DC&T-T1 cutting tool turned out to be 7.69 µm. This highest Ra value was obtained using the DC&T-T1 cutting tool at the applied machining parameters of an 800 rev/min spindle speed and 300 mm/min feed rate. On the other hand, as illustrated in Figure 11a, the lowest Ra value occurring in the holes drilled in the composite workpiece machined with the DC&T-T1 cutting tool turned to be 5.05 µm. This lowest Ra value was obtained using the DC&T-T1 cutting tool at the applied machining parameters of a 2000 rev/min spindle speed and 100 mm/min feed rate. When the highest Ra value (7.69 µm) and the lowest Ra value (5.05 µm) were compared, it was found that the highest Ra value was 52.28% higher than the lowest Ra value. As a matter of fact, when the graph (counter-plot) given in Figure 11b is inspected, it is noticed that the highest Ra values are concentrated (red-colored area) around the machining parameters of an 800 rev/min spindle speed and 300 mm/min feed rate. Additionally, when the graph (counter-plot) given in Figure 11b is inspected, it is noticed that the lowest Ra values are concentrated (blue-colored area) around the machining parameters of a 2000 rev/min spindle speed and 100 mm/min feed rate.

Moreover, as illustrated in Figure 11c, the highest Ra value occurring in the holes drilled in the composite workpiece machined with the T1 cutting tool was found to be 8.99 µm. This highest Ra value was obtained using the T1 cutting tool at the applied machining parameters of an 800 rev/min spindle speed and 300 mm/min feed rate. On the other hand, as seen in Figure 11c, the lowest Ra value occurring in the holes drilled in the composite workpiece machined with the T1 cutting tool was found to be 6.03 µm. This lowest Ra value was obtained using the T1 cutting tool at the applied machining parameters of a 2000 rev/min spindle speed and 100 mm/min feed rate. When the highest Ra value (8.99 µm) and the lowest Ra value (6.03 µm) were compared, it was found that the highest Ra value was 49.09% higher than the lowest Ra value. As a matter of fact, when the graph (counter-plot) given in Figure 11d is inspected, it is noticed that the highest Ra values are concentrated (red-colored area) around the machining parameters of an 800 rev/min spindle speed and 300 mm/min feed rate. Additionally, when the graph (counter-plot) given in Figure 11d is inspected, it is noticed that the lowest Ra values are concentrated (blue-colored area) around the machining parameters of a 2000 rev/min spindle speed and 100 mm/min feed rate.

**Figure 12:**
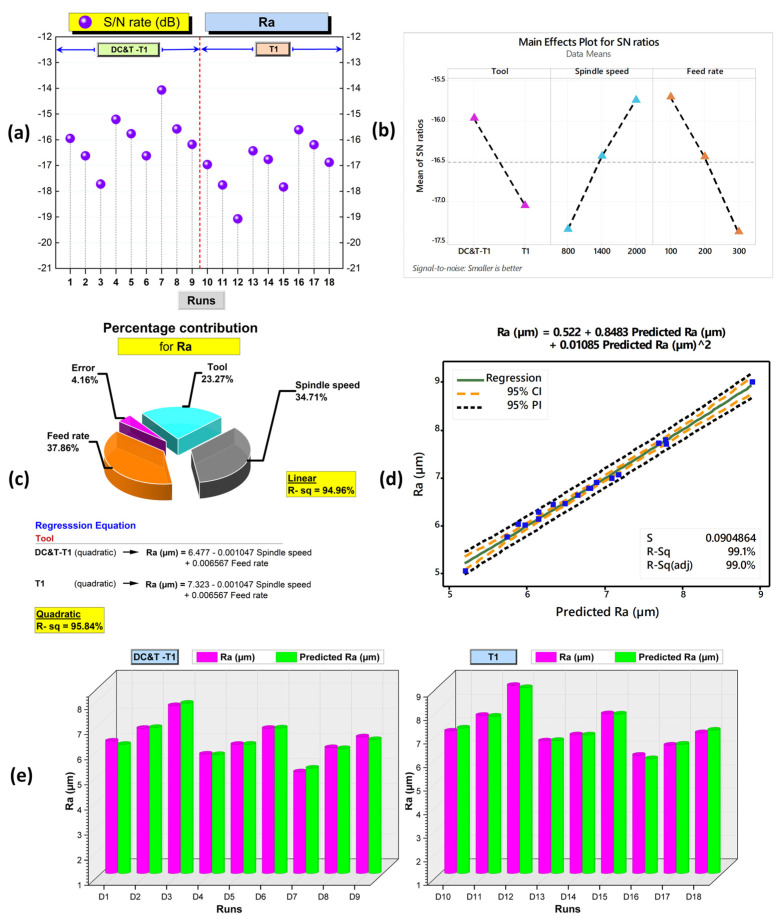
(**a**) Based on Ra values, S/N ratios for each hole. (**b**) Main effects plot for Ra values. (**c**) ANOVA results and regression equations for Ra. (**d**) Comparison of experimental Ra results and predicted Ra values. (**e**) Ra results vs. predicted Ra values.

As a consequence, as depicted in Figure 10 and Figure 11, when Ra values occurring in the holes drilled in the composite workpiece machined (with the same parameters and levels) using the DC&T-T1 and T1 cutting tools were compared, it was observed that better results (i.e., lower Ra values) were obtained with the DC&T-T1 cutting tool. Moreover, when the lowest and highest Ra values obtained with the DC&T-T1 and T1 cutting tools were compared, it was seen that 19.42% and 16.91% better results (i.e., lower Ra values) were obtained with the DC&T-T1 cutting tool, respectively. Furthermore, in this current research, detailed analyses were carried out using the Minitab program in order to better evaluate the Ra values obtained from the holes drilled in the composite workpiece machined using DC&T-T1 and T1 tools. With the help of the Minitab program, S/N ratios were computed based on the Ra values obtained from the holes and displayed in Figure 12a.

As depicted in Figure 12a, the highest S/N ratio (using the Ra value obtained from the hole drilled with the DC&T-T1 cutting tool) was calculated as −14.0658 dB (for an Ra value of 5.05 µm). Additionally, the lowest S/N ratio (using the the Ra value obtained from the hole drilled with the T1 cutting tool) was calculated as −19.0752 dB (for an Ra value of 8.99 µm). Moreover, as illustrated in Figure 12a, although both cutting tools show similar trends depending on the changes in parameters and levels, the DC&T-T1 cutting tool appears to have clear superiority (since a high S/N ratio is desired [31,32]). In this research, in order to better understand the effects of the machining parameters (cutting tool, spindle speed and feed rate), the main effects graph was obtained using S/N ratios and is shown in Figure 12b. When the main effects plot given in Figure 12b is examined, it is clearly seen that better results (i.e., lower Ra values) are obtained with the DC&T-T1 cutting tool. Additionally, as illustrated in the main effects plot (Figure 12b), Ra values are positively affected (i.e., lower Ra values) as the spindle speed increases, while Ra values are negatively affected (i.e., higher Ra values) as the feed rate increases. Furthermore, when the analysis of the S/N ratios (Figure 12b) obtained using Ra values was inspected, it was revealed that the most suitable machining parameters (in order to obtain the lowest Ra values) for the composite workpiece used in this investigation were the DC&T-T1 cutting tool, a 2000 rev/min spindle speed and a 100 mm/min feed rate, respectively. Additionally, within the scope of this current research, separate images of each hole (the highest and lowest Ra values obtained using both cutting tools) were taken in order to better understand the effects of the parameters and levels applied for the machining of the specially manufactured composite workpiece. In Figure 10, the effects of the cutting tools (DC&T-T1 and T1) and cutting parameters on Ra values (i.e., surface roughness) are displayed separately for each hole (for the highest and lowest Ra values). As depicted in Figure 10, the surface roughness values of the holes machined with the DC&T-T1 cutting tool are less than (i.e., smoother) the surface roughness values of the holes machined with the T1 cutting tool. Additionally, it was determined that the Ra values increased with am increasing feed rate at a constant spindle speed in machining operations performed with both cutting tools. On the other hand, it was found that the Ra values decreased with an increasing spindle speed at a constant feed rate in machining operations performed with both cutting tools. Moreover, when the holes machined with both cutting tools (DC&T-T1 and T1) were inspected, it was observed that the Ra values started to decrease (i.e., smoother) significantly as the optimal cutting parameters were approached. As a matter of fact, this situation is better seen in the cutting parameters where a spindle speed of 2000 rev/min and a feed rate of 100 mm/min are applied. Furthermore, when Figure 10, Figure 11 and Figure 12b are considered together, it is observed that the experimental and statistical results are quite compatible.

As is known, by reducing or completely eliminating the retained austenite ratio in the microstructure of cutting tools (HSS tools), significant increases (i.e., improvements) in the hardness, toughness and wear resistance of the cutting tools occur. Moreover, cryogenic treatment is reported to improve the electrical and thermal conductivities of cutting tools [21,23]. Consequently, all these desired, favorable improvements (with the influence of DC&T processes) positively affect the performance of cutting tools [15,22].

As depicted in Figure 10 and Figure 11, it is observed that Ra values increase as the feed rates increase (from 100 mm/min to 300 mm/min) in the holes drilled with both cutting tools (DC&T-T1 and T1). This can be explained by causes such as the increase in the volume of removed chips due to the increase in the feed rate, the inability of the active cutting process to take place and the deterioration of surface integrity (drilled surfaces of the composite workpiece). Moreover, since the thermal conductivity of the composite workpiece used in this research is quite low, the temperature generated during the interaction between the composite workpiece and the cutting tool cannot move away from the area where the cutting process takes place; thus, the composite workpiece softens and positively affects the cutting process [6,41,43,50]. As a matter of fact, when similar studies (investigations carried out on composite workpieces) in the literature were inspected, it was revealed that the Ra values (i.e., less surface roughness) obtained in drilling operations performed with low feed rates were parallel to the Ra values obtained within the scope of this research [44,45]. Additionally, as the feed rate increases, the cutting process cannot be fully performed due to the increase in the volume of the chip that the cutting tool tries to remove from the composite material, which has a negative effect on the Ra values. Here, the cutting tool dives into the composite material faster due to the increase in the feed rate, but since the cutting process cannot be performed fully, it starts to pull and tear the composite material instead of cutting it. On the other hand, as displayed in Figure 10 and Figure 11, it is observed that Ra values decrease as the spindle speed increases (from 800 rev/min to 2000 rev/min) at a constant feed rate in the holes drilled with both cutting tools (DC&T-T1 and T1). This can be explained by the fact that less surface roughness occurs on the hole due to the better active cutting process (during the interaction between the cutting tool and the composite workpiece) in drilling operations performed at high spindle speeds. Furthermore, due to the positive effect of the high spindle speed, high temperatures occur in the composite material (due to its low thermal conductivity); consequently, the cutting tool performs the active cutting process more easily, and thus, the surface roughness caused by the drilling process begins to occur at minimum values [6,41,43,46]. As a matter of fact, when similar studies (investigations carried out on composite workpieces) in the literature were inspected, it was noticed that the Ra values (i.e., less surface roughness) obtained in drilling operations performed with high spindle speeds were parallel to the Ra values obtained within the scope of this research [2,41,43,47,50].

#### 3.2.1 ANOVA (Analysis of the Variance) and Regression Analyses Based on Ra Values

In this current investigation, ANOVA was carried out at 95% confidence and 5% significance levels to find the percentage contribution values (in order to better understand the effect of each factor on the Ra values) of the cutting tools and cutting parameters [31,32]. In Figure 12c, the results of the ANOVA conducted based on the Ra values are visualized. As illustrated in Figure 12c, when the ANOVA results acquired based on the Ra values obtained for each hole within the scope of this experimental investigation were examined, it was discovered that the most significant parameter having an impact on Ra values was the feed rate, with a rate of 37.86%. Additionally, the percentage contribution rates of other parameters with an influence on the Ra values were found to be 34.71% (spindle speed) and 23.27% (tool), respectively. As a matter of fact, when the pictures taken at the hole edges given in Figure 10 and 11 are inspected, it is seen that they confirm the ANOVA results. Additionally, as illustrated in Figure 12c, regression analyses were carried out based on the Ra values obtained within the scope of this research. With regression analysis, the correlation between process parameters and the responses of any machining process is analyzed in detail, and high-accuracy regression equations are developed [31,32]. As depicted in Figure 12c, as a consequence of the analyses performed according to the Ra values, separate equations for the DC&T-T1 and T1 cutting tools were formulated as quadratic and linear models. When the regression coefficients (R-sq, that is, R2) indicating the effectiveness of the developed models [32] were examined (Figure 12c), it was revealed that the R2 value (95.84%) of the quadratic model was higher than the R2 value (94.96%) of the linear model. The high regression coefficients (90% and above) obtained show that the equations developed for the DC&T-T1 and T1 cutting tools are highly successful in predicting Ra values [31,32]. In Figure 12d, the comparison of the Ra values predicted using the highly successful quadratic regression model with the experimentally obtained Ra values is illustrated. When the graphic (Figure 12d) comparing the experimental results with the predicted values is visually examined, it is discovered that the outcomes (blue squares) are gathered quite close to the green line (regression line). Considering these results obtained, it is found that the developed model is highly successful and reliable in estimating Ra values [32,49]. Furthermore, each experimental result and the predicted value are compared separately (with the aim to demonstrate the high success of the developed model) and displayed in Figure 12e. When the experimental Ra results and predicted Ra values given in Figure 12e are visually examined, it is seen that the experimental Ra results and predicted Ra values are equal or very close to each other. In light of these results, it is clearly seen that the developed model (for the purpose of predicting Ra values) is highly reliable and successful.

## 4. Conclusions

In this experimental research, the performances of specially manufactured cutting tools (cryo-treated and untreated cutting tools) for high-quality machining of eco-friendly jute fiber-reinforced epoxy composite workpiece were investigated. The experimental and statistical results obtained from this research are presented below separately.

As a consequence of the DC&T processes applied to the cutting tool, the average hardness value (798.3 HV) of the cutting tool (DC&T-T1) subjected to DC&T processes was found to be 13.89% higher than the average hardness value (700.9 HV) of the untreated cutting tool (T1). Thus, it was observed that the applied DC&T processes provide the necessary improvements that positively affect the performance of the cutting tool.

When the lowest and highest Fd values obtained with DC&T-T1 and T1 cutting tools were compared, it was observed that 5.49% and 6.23% better results (i.e., lower Fd values) were obtained with the DC&T-T1 cutting tool, respectively. Additionally, it was seen that the Fd values increased as the feed rates increased in the holes drilled with both cutting tools (DC&T-T1 and T1). On the other hand, it was found that Fd values decreased as the spindle speed increased in the holes drilled with both cutting tools. From the analysis of the S/N ratios obtained using the Fd values, it was found that the most suitable machining parameters (in order to obtain the lowest Fd values) for the composite workpiece used in this investigation were the DC&T-T1 cutting tool, a 2000 rev/min spindle speed and a 100 mm/min feed rate, respectively. In the ANOVA conducted, it was discovered that the most significant parameter having an impact on Fd values was the spindle speed, with a rate of 53.01%. Additionally, as a consequence of the regression analyses performed for the Fd values, the R2 values (based on the linear and quadratic models developed) were found to be quite high (i.e., 90% and well above).

Considering the lowest and highest Ra values obtained using the DC&T-T1 and T1 cutting tools, it was seen that 19.42% and 16.91% better results (i.e., lower Ra values) were obtained using the DC&T-T1 cutting tool, respectively. Additionally, it was determined that during the drilling of the composite workpiece with both cutting tools (DC&T-T1 and T1), the Ra values decreased (i.e., positively affected) as the spindle speed increased, but the Ra values increased (i.e., negatively affected) as the feed rates increased. From the analyses of the S/N ratios obtained using Ra values, it was revealed that the most suitable machining parameters (in order to obtain the lowest Ra values) for the composite workpiece used in this investigation were the DC&T-T1 cutting tool, a 2000 rev/min spindle speed and a 100 mm/min feed rate, respectively. In the ANOVA conducted, it was discovered that the most significant parameter having an effect on Ra values was the feed rate, at 37.86%. Furthermore, as a consequence of the regression analyses performed for the Ra values, the R2 values (based on the linear and quadratic models developed) were found to be quite high (i.e., well above 90%).

## Figures and Tables

**Table 1:** Machining parameters and levels.

Machining Parameters and Levels				
Parameter	Unit	Level 1	Level 2	Level 3
Spindle speed	rev/min	800	1400	2000
Feed rate	mm/min	100	200	300
Tool	-	DC&T-T1	T1	-

**Table 2:** Machining parameters and levels.

Runs	Tool	Spindle Speed (rev/min)	Feed Rate (mm/min)
D1	DC&T-T1	800	100
D2	DC&T-T1	800	200
D3	DC&T-T1	800	300
D4	DC&T-T1	1400	100
D5	DC&T-T1	1400	200
D6	DC&T-T1	1400	300
D7	DC&T-T1	2000	100
D8	DC&T-T1	2000	200
D9	DC&T-T1	2000	300
D10	T1	800	100
D11	T1	800	200
D12	T1	800	300
D13	T1	1400	100
D14	T1	1400	200
D15	T1	1400	300
D16	T1	2000	100
D17	T1	2000	200
D18	T1	2000	300

## Data Availability

The data involved in the findings of the result will be shared by the corresponding authors based on reasonable request.
